# *Atmiyata,* a community-led intervention to address common mental disorders: Study protocol for a stepped wedge cluster randomized controlled trial in rural Gujarat, India

**DOI:** 10.1186/s13063-020-4133-6

**Published:** 2020-02-21

**Authors:** Kaustubh Joag, Jasmine Kalha, Deepa Pandit, Susmita Chatterjee, Sadhvi Krishnamoorthy, Laura Shields-Zeeman, Soumitra Pathare

**Affiliations:** 1 Centre for Mental Health Law and Policy, Indian Law Society, Law College Road, Pune, 411004 India; 2 George Institute for Global Health, Elegance Tower, 311-312, Third Floor, JasolaVihar, New Delhi, Delhi 110025 India; 3 Netherlands Institute for Mental health and Addiction (Trimbos Institute), Da Costakade 45, 3521 VT Utrecht, the Netherlands

**Keywords:** Stepped wedge cluster randomised controlled trial, Community-led intervention, Community mental health, Low-and-middle-income countries, Evidence-based practice, Common mental disorders

## Abstract

**Background:**

While lay-health worker models for mental health care have proven to be effective in controlled trials, there is limited evidence on the effectiveness and scalability of these models in rural communities in low- and middle-income countries (LMICs). *Atmiyata* is a rural community-led intervention using local community volunteers, called Champions, to identify and provide a package of community-based interventions for mental health, including evidence-based counseling for persons with common mental disorders (CMD).

**Methods:**

The impact of the *Atmiyata* intervention is evaluated through a stepped wedge cluster randomized controlled trial (SW-CRCT) with a nested economic evaluation. The trial is implemented across 10 sub-blocks (645 villages) in Mehsana district in the state of Gujarat, with a catchment area of 1.52 million rural adults. There are 56 primary health centers (PHCs) in Mehsana district and villages covered under these PHCs are equally divided into four groups of clusters of 14 PHCs each. The intervention is rolled out in a staggered manner in these groups of villages at an interval of 5 months.

The primary outcome is symptomatic improvement measured through the GHQ-12 at a 3-month follow-up. Secondary outcomes include: quality of life using the EURO-QoL (EQ- 5D), symptom improvement measured by the Self-Reporting Questionnaire-20 (SRQ-20), functioning using the World Health Organization’s Disability Assessment Scale (WHO-DAS-12), depression symptoms using the Patient Health Questionnaire (PHQ-9), anxiety symptoms using Generalized Anxiety Disorder Questionnaire (GAD-7), and social participation using the Social Participation Scale (SPS). Generalized linear mixed effects model is employed for binary outcomes and linear mixed effects model for continuous outcomes. A Return on Investment (ROI) analysis of the intervention will be conducted to understand whether the intervention generates any return on financial investments made into the project.

**Discussion:**

Stepped wedge designs are increasingly used a design to evaluate the real-life effectiveness of interventions. To the best of our knowledge, this is the first SW-CRCT in a low- and middle-income country evaluating the impact of the implementation of a community mental health intervention. The results of this study will contribute to the evidence on scaling-up lay health worker models for mental health interventions and contribute to the SW-CRCT literature in low- and middle-income countries.

**Trial registration:**

The trial is registered prospectively with the Clinical Trial Registry in India and the Clinical Trial Registry number- CTRI/2017/03/008139. URL http://ctri.nic.in/Clinicaltrials/regtrial.php?modid=1&compid=19&EncHid=70845.17209. Date of registration- 20/03/2017.

## Background

Mental illness is a substantial public health burden in India, affecting 10.6% of the population [1]. There is a shortage of mental health professionals to address the mental health needs of the population, particularly in rural areas [1]. This is further compounded by a high level of public stigma towards mental illness and the lack of accessible mental health care, resulting in a treatment gap of nearly 80–90% for mental illness in India [1].

In most low- and middle-income countries (LMICs), service delivery models have focused on task-sharing, the process of sharing mental health care tasks with less specialised health workers, such as community health workers [2]. Several programs have been developed to build the capacity of community members and primary care health workers, with the aim of increasing their uptake of mental health tasks and enable access to mental health supports in rural areas [3, 4]. A number of studies in India and other parts of South Asia have shown the efficacy of task-sharing initiatives [5–8].

A possible reason for the inability to scale-up task-sharing models may be that public health systems in low-and-middle-income countries (LMICs) are overburdened with addressing other health needs, and there are limited time and energy to devote to mental health [9–12]. Task sharing approaches may, therefore, need to build community capacity to provide mental health care that complements service provision efforts within the public health system. *Atmiyata* is a community-led intervention [13] using non-specialised community volunteers for identification, support, and referral for persons with common and severe mental disorders. *Atmiyata* was previously piloted in 41 villages in the Nashik district of the state of Maharashtra from 2013 to 2015 [13].

As a mental health program, *Atmiyata* aims to: (i) reduce the treatment gap for common and severe mental disorders; (ii) improve mental health outcomes for people with common mental disorders (CMD); (iii) improve quality of life among people with mental health problems; (iv) improve access to social welfare schemes for people with mental health problems. We hypothesize that the intervention will result in symptomatic improvement in CMD (depression and anxiety) and well-being as well as narrow the mental health ‘care’ gap [14]. Evaluating both the efficiency as well as the implementation process of this intervention will generate valuable lessons as to how we might sustain the intervention’s impact when delivered to a large population.

## Methods

### Design

We employ a Stepped Wedge Cluster Randomized Controlled Trial (SW-CRCT), with a nested health economic evaluation to assess the impact on persons with CMD and return on investment (ROI) of the intervention in the Indian state of Gujarat. The stepped wedge design is chosen for evaluating the scale-up of *Atmiyata* intervention as it allows for random allocation of the timing in which clusters receive the intervention [15]. All clusters receive the intervention before the trial ends which is ethically appropriate. The design also allows for *Atmiyata* intervention to be delivered in a staggered manner to account for practical logistics constraints; it is not feasible to deliver the intervention in all clusters (villages served under groups of primary health centers) simultaneously. Stepwise implementation allows the implementation team enough preparation time and is an efficient use of implementation team resources. Additionally, the staggered implementation of the intervention over time periods allows for more in-depth statistical analysis compared to a pre-post, parallel-arm cluster randomized controlled trial design.

**Fig. 1:**
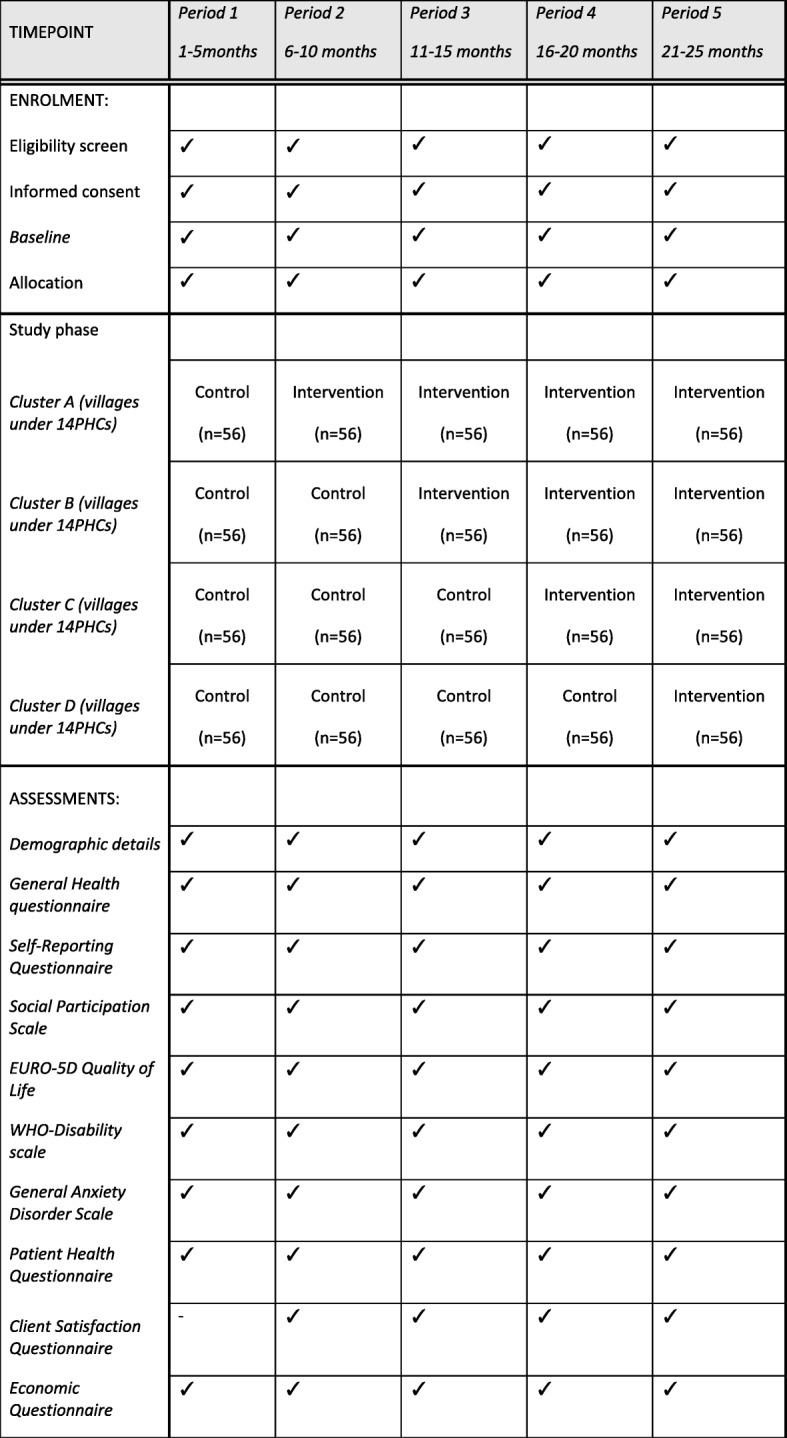
SPIRIT figure. Enrollment, allocation, intervention, and assessment were taken place in all periods as this is a cross-sectional SW-CRCT

There are 56 primary health centers (PHCs) in the Mehsana district (where the study takes place), and each PHC serves discrete villages within a geographical area. Each village in the geographical area served by a PHC is a cluster in this study. We created 4 groups of clusters (A, B, C, D), each made up of villages covered by 14 PHCs. Groups are created according to geographical location to help reduce the probability of contamination between groups; villages in Group A are farther from Group B villages and villages in Group C are farther from Group D. All groups (A, B, C, D) are allocated to intervention condition at different steps. A ‘Step’ is the order in which a group of clusters switches from control to intervention condition. On the other hand, ‘Period’ is defined as a group of observations by the time of measurement. The duration of each period is 5 months to accommodate for baseline and 3 months of follow-up data collection (Fig. 1).

This study uses a repeated cross-sectional design with outcome data derived from different participants in each period. All four clusters start at baseline in the control condition and are exposed to the intervention at a regular period of five months (Fig. 1).

### Setting

The intervention is implemented in Mehsana district, located in Western India in the state of Gujarat. It is primarily a rural district (75% rural), with a rural population of 1.52 million people, of which approximately one million are above 18 years of age. The district is divided into 10 blocks/ sub-districts with a total of 645 villages and 316,536 rural households [16]. Almost half (45.4%) of Mehsana’s rural population has a low standard of living as defined by the Standard of Living Index [17]. Most residents (53%) are employed in the agricultural sector. The rural population of the Mehsana district is economically disadvantaged as agriculture is not always a viable occupation given uncertain climatic events [16]. In terms of health services, Mehsana has 56 PHCs, 11 Community Health Centers and 1 District Hospital staffed by 2 psychiatrists, along with District Mental Health Program (DMHP) which provides additional human resources (such as a psychologist and social worker) for mental health at the community level. Mental health care is primarily delivered by psychiatrists at the District Hospital and the psychiatrists also visit Community Health Centers on a fortnightly basis in rotation, as part of DMHP. The district hospital has in-patient and out-patient services for persons with mental illness, and limited psychosocial support services.

### Participants

The study sample consists of adult community members with CMD (e.g. anxiety and depression) residing in rural villages in the Mehsana district.

#### Inclusion Criteria

- ▪ Persons aged 18 years or more and less than 65 years of age
- ▪ A Score of 3 or above on the General Health Questionnaire (GHQ-12), indicating a case with CMD

#### Exclusion criteria

- ▪ Persons who cannot provide informed consent or decline participation in the study
- ▪ Persons with a terminal medical condition
- ▪ Persons who have suicidal ideation or plans for suicide at the baseline interview

### Primary outcome

The primary outcome is the symptomatic improvement of CMD as measured using a validated Gujarati version of the GHQ-12 [18] from baseline to 3-month and 8-month follow-up to evaluate sustained effects of the intervention. The GHQ-12 is a widely used screening tool with reliable sensitivity for assessing CMD [19]. GHQ-12 is a dichotomous 12-item questionnaire with each item rated on a 4-point scale, with possible responses being “less than usual,” “no more than usual,” “rather more than usual,” or “much more than usual.” We used a bimodal scoring method, whereby “less than usual” and “no more than usual” is scored as 0 point, and “rather more than usual” and “much more than usual” is scored as 1 [18]. GHQ-12 scores will be analyzed as both continuous (ranging from 0 to 12) and categorical outcomes (case defined as 3 and above scores on GHQ scale; non-case as less than 3 scores on GHQ scale).

### Secondary outcomes

Secondary outcome measures are assessed at 3 and 8 months after the start of the intervention (Table 1).

**Table 1:** Follow-up assessment of tools with time points

ASSESSMENT TOOLS	Baseline	3-month follow-up	8-month follow-up
*Demographic information*	✓	✓	✓
*General Health questionnaire*	✓	✓	✓
*Self-Reporting Questionnaire*	✓	✓	✓
*Social Participation Scale*	✓	✓	✓
*EURO-5D Quality of Life*	✓	✓	✓
*WHO-Disability scale*	✓	✓	✓
*General Anxiety Disorder Scale*	✓	✓	✓
*Patient Health Questionnaire*	✓	✓	✓
*Client Satisfaction Questionnaire (only in intervention condition)*	–	–	✓
*Economic Questionnaire*	✓	✓	✓

#### Quality of life

Improvement in quality of life among persons with CMD is assessed using a validated Gujarati version of EURO Quality of life- 5D (EQ-5D) [20]. The EQ-5D’s descriptive system is a preference-based Health Related Quality of Life measure with one question for each of the five dimensions that include mobility, self-care, usual activities, pain/discomfort, and anxiety/depression measured at 5 levels: no problems, slight problems, moderate problems, severe problems, and extreme problems. A lower score indicates a better quality of life [20].

#### Psychiatric symptoms

Improvement in psychiatric symptoms is assessed using a validated Gujarati version of the Self Reporting Questionnaire (SRQ). SRQ is a scale developed by the World Health Organization to screen for psychiatric disturbances for low- and middle-income countries. It consists of 20 questions which are scored 1 = yes and 0 = no, indicating the presence or absence of a particular symptom over the past month. SRQ is a continuous scale and responses are calculated as a total score ranging from 0 to 20, with lower scores indicating recovery of symptoms [21].

#### Disability

Reduction of disability and reduction in a number of days unable to work and improvement in productivity is assessed using a validated Gujarati version of the World Health Organization’s Disability Assessment Scale (WHO-DAS-12). The WHO-DAS-12 assesses overall functioning, in relation to difficulties due to health conditions. The scale has 12 items, with a 5-point rating scale ranging from none, mild, moderate to severe and extreme. Responses are calculated as a total score ranging from 12 to 60 [22].

#### Depression and anxiety symptoms

Improvements in depression symptoms are assessed using a validated Gujarati version of the Patient Health Questionnaire (PHQ-9). PHQ-9 scores range from 0 to 27, with a higher score indicating more severe symptoms [23]. Improvements in anxiety scores are assessed by using a validated Gujarati version of Generalized Anxiety Disorder (GAD-7) with total scores ranging from 0 to 21 [24].

#### Social Participation

Increases in social participation is assessed using the Social Participation Scale (SPS) [25]. The SPS is an 18-item interview-based instrument measuring perceived problems in major domains of life such as learning and applying knowledge, communication, mobility, self-care, domestic life, interpersonal interactions, major life areas (like work, life, and employment) and community (like leisure, recreation, political life). The scale allows quantification of participation restrictions experienced by people affected by disability or other stigmatized conditions. The 18 items are rated on a 5-point scale ranging from 0 (no problem) to 5 (large problem). Responses are calculated as a total score ranging from 0 to 90 [26].

#### Service User Satisfaction

User-satisfaction with the intervention is assessed using a validated Gujarati version of the Client Satisfaction Questionnaire (CSQ) [25] at 8-month follow-up. The CSQ is an 8-item scale assessing client satisfaction with care/treatment received. The scoring uses a 4-point rating scale and is scored by summing the individual scores to produce a range of 8 to 32, with higher scores indicating greater satisfaction with care [27].

#### Specificity of identification of CMD cases

The accuracy of Champions identifying community members with CMD by Champions is assessed using the GHQ-12.

### Economic Evaluation

The most common measure of efficiency of interventions in the health sector is cost-effectiveness analysis (CEA) which measures health-related benefits and expresses these in a natural unit such as lives saved, or symptoms reduced. A return-on-investment analysis, however, expresses benefits in monetary terms relative to investments made. Expressing both the costs and the full range of benefits of an intervention in the same units (money) has the distinct advantage of making investment decisions very straightforward [28]. If the money value of the benefits of an intervention is larger than the cost of the intervention, it may be regarded as a sound investment. Hence, we have chosen to do ROI analysis for the *Atmiyata* intervention.

Costs are calculated using both government and societal perspective [29]. Perspective determines the cost components to be included in any cost analysis and societal perspective is the broadest viewpoint that covers all costs irrespective of who incurs these costs. On the other hand, a government perspective only includes costs incurred by the government for a particular health intervention. Costs are accounted under two categories: 1. total cost of the intervention and; 2. treatment cost of CMDs. Treatment costs are categorized as: direct medical cost, direct non-medical cost, and indirect costs. Direct medical costs include out of pocket expenses incurred in order to seek treatment (e.g. diagnostic tests, fees for consultation in clinics, traditional healers, hospitals, bed day charges at a public or private health facility). Direct non-medical costs include the amount spent for travelling to the health facility for the patient and accompanied persons for treatment, the amount spent on meal/food taken while waiting for treatment, expenses for overnight accommodation for seeking care, etc. Indirect costs represent the opportunity cost for the patient and their household members’ time related to CMD. Cost data is collected at baseline, 3 months and 8 months from all study participants. Time spent by the champion will be obtained from the program implementation data. The minimum wage rate of Gujarat will be used to value their time. Total hours spent on the program will be multiplied by hourly wage (obtained from the minimum wage rate) to get the time cost of the Champions. Benefits are considered in terms of improved health, functioning, participation, productivity, increased saving and investment, reduced informal care giving and health and welfare services. Lost workdays before and after the intervention will be obtained through the WHO-DAS 12 questionnaire and will be linked to the minimum wage rate to estimate the aggregate effect. Being alive and healthy is also considered valuable and the overall value of a life year can be broken down into its economic (instrumental) and health (intrinsic) elements. Following the approach used in prior ROI analyses in mental health, we use the figure of 0.5 times the per-capita income of India as the value of a healthy life year [28, 30, 31].

### Intervention condition

The *Atmiyata* intervention has been described extensively elsewhere [13]. Briefly, *Atmiyata* is a complex psychosocial intervention involving two-tiers of community volunteers for identification and support to people in distress and with symptoms of common mental disorders. The first tier consists of community volunteers called *Atmiyata* Mitras who are from different caste and religion-based sections of the village, trained to identify persons in mental distress. The second tier consists of *Atmiyata* Champions, who are important community members (e.g. former teachers, community leaders) with leadership and communication skills and are well-known and approachable in their village. Champions are trained to identify and provide structured counseling to persons with significant mental distress, including the ones referred by Mitras. Given the social barriers based on caste, gender, religion, the identification and support by Champions and Mitras ensure equitable reach and improves coverage of the intervention across the entire village.

**Fig. 2:**
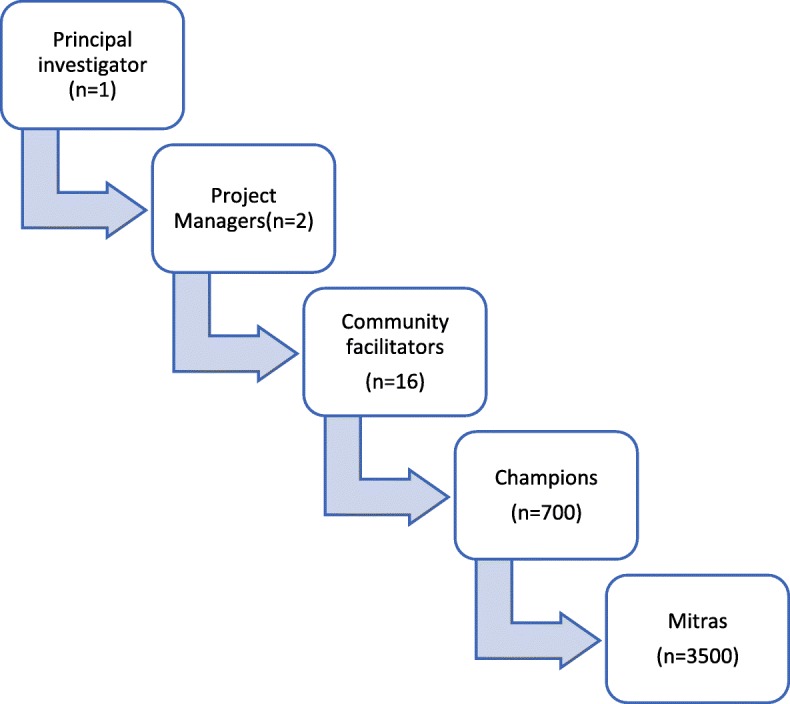
*Atmiyata* Implementation team structure

In the *Atmiyata* pilot intervention in the state of Maharashtra in 2013–2015, Champions and Mitras were trained by the project team. In this study, the *Atmiyata* Gujarat program, where the target population is substantially larger, Champions are identified and trained by Community Facilitators (CF) who typically have a master’s degree in social work or related fields, are locally based and aware of community dynamics. CFs first map their allotted villages, then identify and recruit the Champions, train them and provide ongoing mentoring support to Champions. The CFs are recruited, trained and mentored by Project Managers (PM). Each PM supports 7–8 CFs. Each CF supports 40–50 Champions (1 per 1000 population), and each Champion has 4–5 Mitras. The principal Investigator (PI) monitors and supports the PMs (Fig. 2).

Mitras receive 4 h of training from a Champion based in a particular village. Champions receive 40 h of training over 3 weeks, at a central location in the block or village area. PMs and CFs receive 55 h of training over 5 weeks, with additional 8-h sessions on how to be a master-trainer. The methodology of the training is interactive, reflective and participatory.

The Champions are trained: (i) to identify persons with CMD and provide evidence-based 4–6 counseling sessions; (ii) to raise community awareness on social issues by ‘narrow-casting’ four films; 10-min films dubbed in Gujarati on commonly experienced social issues in the community such as unemployment, family conflict, domestic violence, and alcoholism. Films are developed to build community mental health awareness and were not designed for training purposes or for intervention delivery related to counselling or symptom reduction. The films are shown to community members in the village where the Champions reside, typically in a public space in the village. Champions show these films in a small group of 3 to 4 people at communal meeting places in a village such as a temple, at a farm or Champion’s house [32].; Champions also (iii) make referrals of persons with severe mental disorders to mental health services offered within the public health system when required, and (iv) enable access to social benefits for persons with mental health problems, such as government schemes for paid work opportunities.

Counseling sessions by Champions are based on three evidence-based techniques used in prior and similar task-sharing programs [33, 34]: active listening, activity scheduling and problem-solving techniques. The training includes basic skills of empathy, non-judgemental behaviour, rapport building, verbal and non-verbal communication and creating a safe environment to build skills for ‘active listening’. Activity scheduling techniques enable individuals to explore reasons why they have been avoiding activities and schedule pleasurable and valuable activities to resume day to day routine [35, 36]. Problem-solving techniques are used for a positive orientation towards the problem which enables viewing problems as solvable, and as opportunities to learn and change. Champions are trained to deliver 4–6 sessions of counseling over a period of 6 to 12 weeks, with no set time between sessions. Each session lasts 20 to 40 min based on mutual agreement between the Champion and the participant. The number of sessions delivered to the participant is also left to mutual agreement between the Champion and the participant. As this is an implementation research study, we aim to assess what happens when the intervention is implemented in ‘real-life’ settings where the number, duration, and frequency of sessions will vary between the different provider (Champion) and participant dyad. We therefore provide broad guidelines and recommendations on the number, duration and frequency of sessions to Champions (e.g. a range of sessions from 4 to 6 sessions, duration from 20 to 40 min, and period of 6–12 weeks) which both mimics what is likely to happen in real-life clinical settings and allows us to explore the possibility of dose-response effects in the study. Champions deliver the intervention at the participant’s home or immediate surroundings at a location preferred by the participant (e.g. champion’s home, a community place such as village hall, temple, etc.). Thus, participants do not have to travel to receive the intervention. The delivery of intervention is in the Gujarati language.

Supervision and mentoring are important components of our Intervention [13]. In the *Atmiyata* intervention, the PI (who is also a clinician) is the primary supervisor of the PMs and CFs. The PMs and CFs supervise and mentor champions delivering the intervention. The PMs and CFs receive 7 days of training in the intervention with an additional 3 days of training on supervision and mentoring. These 10 days of training for CFs and PMs are supplemented through routine supervision discussions with the PI. The PMs and CFs go on to conduct a 7-day training for the Champions. CFs visit the Champions on-site once a month for hands-on mentoring sessions. During these visits, CFs discuss with Champions the counseling sessions delivered by them, check the structure of each session and troubleshoot any difficulties in using counseling techniques. CFs also help champions clarify concepts and if necessary, demonstrate counseling techniques. These monthly visits ensure the quality and fidelity of the intervention are maintained. Day-long refresher sessions are scheduled once every three months to bring Champions and CFs back together to discuss common challenges and strategies to overcome challenges in delivering the intervention.

### Adverse events

We considered adverse events as attempted suicide, self-harm or death by suicide. A protocol for reporting and recording of adverse events is provided in the Additional file 1. All team members are trained by the data manager and PI on proper identification, recording, and reporting of adverse events. All adverse events are tracked as per the protocol and reported to the Institutional Ethics Committee within 15 days of occurrence. We decided to only track the above-mentioned adverse events because *Atmiyata* provides low-intensity counseling package delivered by Champions to people with distress and CMD, and other events such as non-suicide related hospitalizations are not directly related to the service being provided. This specific definition of adverse events was agreed upon by the ethics committee advising the project on ethical considerations and documented and defined in the trial protocol.

### Comparison condition

Participants in the control condition receive Enhanced Usual Care (EUC). EUC is offered to all participants in the comparison condition who scored 3 and above on GHQ-12. EUC provides information on the impact of distress on their physical and mental health and relevant information of accessible and available public mental health care services, including services by the District Mental Health Programme (DMHP), and helplines for mental health support and domestic violence in and around Mehsana district.

The EUC conditions also has provisions for providing active support to participants in crisis. A crisis is defined as the participant revealing a recent self-harm attempt or expressing thoughts of self- harm during data collection. Such participants are encouraged to seek help immediately and the data collectors seek participant consent to inform their family member or a friend about the crisis and thus, mobilize social support to deal with the crisis.

### Sample size and power calculations

**Fig. 3:**
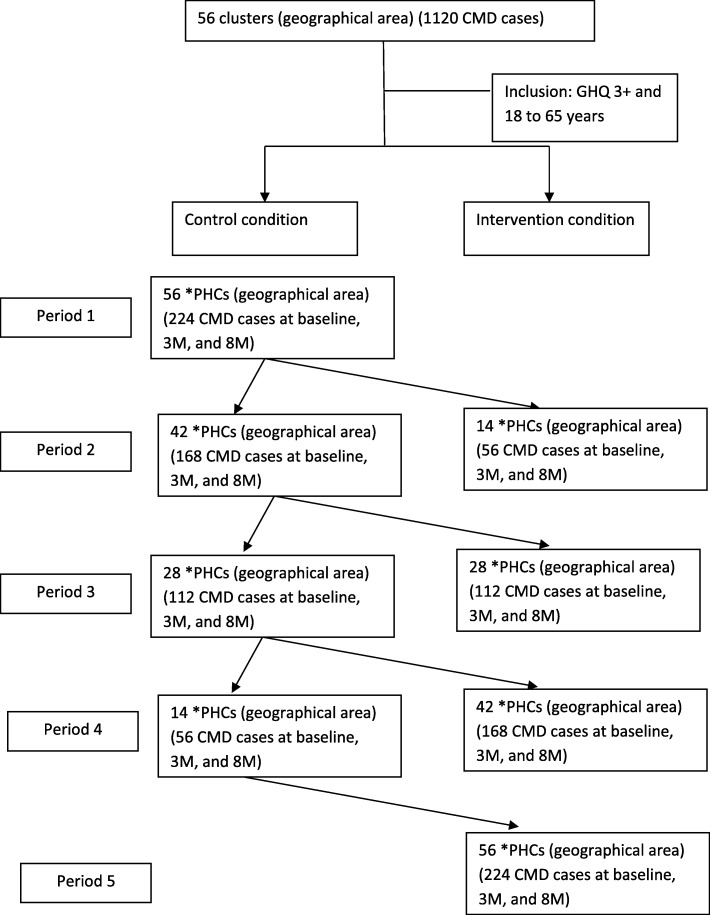
CONSORT Flow chart for *ATMIYATA* SW-CRCT design *PHCs in the consort diagram refers to groups of clusters based on geographical area

A trained lay health worker-led intervention study conducted in India reported a risk difference of 12% at follow up between intervention and control condition for the recovery of CMD patients [5, 37]. The sample size for this SW-CRCT is calculated to detect a 13% difference in CMD cases at a 3-month follow-up using GHQ-12 as a categorical measure between the intervention (58% improved) and control condition (45% improved). Assuming an intra-cluster correlation coefficient of ICC = 0.1, number of steps (t = 4), number of clusters randomized in each step (k = 14), average cluster size (m = 4), power (80%) and alpha of 0.05, a sample size of 1120 participants is needed, with approximately 56 individuals per cluster per period (Fig. 1, 3). The sample size was calculated using the “stepped wedge” function of STATA version 14 [38].

### Randomization and treatment allocation

The unit of randomization in stepped wedge trials is a cluster or group of clusters, allocated to different steps. In India, PHCs serve discrete villages and hence we have used the PHC as a unit to identify discrete geographical areas consisting of all villages under a PHC. This geographical area is taken as a cluster for our study. Typically, a PHC in Gujarat covers a population of 25,000–30,000 across 12–13 villages. There are 56 PHCs in the Mehsana district, split into 4 groups (A, B, C, D), with each group consisting of all villages under 14 PHCs. These groups of clusters (A, B, C, D) are sequentially allocated to different steps at given time periods. Randomization takes place at the level of participants, all the participants are randomly selected for each period from each of these groups of clusters (A, B, C, D) as described in the recruitment section below. The administrative organization of primary health care in Gujarat ensures that the inter-PHC movement of people for health care is minimal and avoids contamination of the intervention. Since double-blinding is not possible in such psychosocial intervention trials, several other procedures are used to minimize contamination and bias. To minimize contamination due to the intervention and control village members meeting and potentially discussing the intervention, clusters are geographically dispersed. Second, the data collection team is separate from the intervention team and blind to the treatment status. Third, the data collection staff receives initial training and re-training at repeated intervals to ensure the quality of data collected.

### Recruitment

In the control condition (enhanced usual care), a screening list is generated from the district electoral roll using a systematic random sampling method with pre-decided random start and random interval, with every *n*th number from the pool being selected. We used electoral roll as it is the most complete, comprehensive and accessible national frame of residential addresses in India and electoral rolls are extensively used for drawing a random sample from the general population [39]. For each group of clusters (A, B, C, and D) electoral rolls from all the villages under 14 PHCs are included. Since the prevalence of CMD is 4–8%, according to the National Mental Health Survey, India [1], a screening list using a minimum prevalence of 4% and assuming 25% missing persons is prepared. For each group (A, B, C and D) in each period of the control condition, a screening list of 1800 participants is created and screened (GHQ-12 score of 3 and above) to achieve the target sample of 56 participants.

A different recruitment procedure is used in the intervention condition, as using structured questionnaires for identification (e.g. GHQ, PHQ) was perceived as impractical when implementing the intervention at scale and seen as stigmatizing in a community setting. Champions are trained to identify a person in their catchment area (i.e. villages) with CMD based on symptoms described by the participant during an unstructured interview. When a Champion identifies a person with CMD to whom they intend to provide 4–6 counseling sessions to, they are asked to inform their CF who in turn informs the data manager who creates a caseload list for each Champion. All caseload lists across Champions are then merged to create a master list. The master caseload list has the residential address of the persons identified by the Champion, who are then approached by the data collection team. Champions obtained verbal consent from each person before providing any personal information such as the address to the CF. CFs are trained to maintain confidentiality of this information (see the section on data storage, confidentiality below). The sample for the intervention condition is drawn from this master caseload list using a computer-generated random method. The drawn sample is screened by data collection staff using GHQ-12 (score of 3 and above) for recruiting intervention participants. This screening and subsequent baseline data collection for participants meeting the inclusion criteria is done prior to the Champion starting psychosocial counseling sessions with the participant. This process is continued until the target sample size of 56 cases is reached for each cluster and period. A different recruitment procedure for the intervention condition was chosen in order to estimate the specificity (accuracy) of ident ification of CMD cases by Champions, which is one of the secondary outcomes. Using similar recruitment procedures in both control and intervention condition (using electoral rolls) would have answered the coverage question (how many people with CMD in the community were identified by the Champion) but not allow us to assess accuracy of identification. We first want to establish whether Champions are accurately able to identify persons with CMD, before addressing the coverage question. Furthermore, we have other data to estimate the population coverage of the intervention.

### Data collection

Written Informed consent is sought from all participants. A thumb impression and signature of a witness is taken for illiterate participants [40]. Data is collected by trained researchers in two stages using a paper-pencil method. In the first stage, demographic data along with GHQ-12 is collected. The data collectors score the GHQ-12 using a scoring sheet and if the participant has a score of 3 or more, secondary outcome data is collected at the same time. The questionnaire for secondary outcome data takes 40–45 min to complete. Participants are not compensated for their time, as this is a volunteer-led intervention. Data collection staff travel to participant’s home for data collection to avoid any travel costs for participants. Data collectors are recruited from the intervention district but recruited from different villages. Data collectors are not paired (based on age, gender, caste, and religion) with participants and they are not assigned participants from their villages for data collection, thus reducing the likelihood of study staff having prior acquaintance with the participants. Furthermore, if any study staff had any prior personal acquaintance with a participant, they were replaced with another data collector who was not acquainted with the participant.

### Data management

*Atmiyata* uses a comprehensive data management system that aids in collecting high-quality data by maintaining on-going- on-site and off-site quality assurance and quality control checks. Research staff (data collectors) handling data are thoroughly trained in interview techniques and procedures for sensitive data handling. Several measures to control for quality of data collected are implemented, including weekly checks, field monitoring visits twice a month by data manager and spot checks (once a month). Additionally, refresher training sessions are provided once in 4 months for quality assurance purposes. The data manager ensures completeness and legibility of the data prior to data entry and is responsible for storing all the data. A designated data entry person is trained for specific entry guidelines to avoid erroneous data entry. The de-identified data is entered in a password-protected Excel sheet. Personally identifiable information is not entered in the database. Raw data is not uploaded on the internet; instead all entered data is shared with the statistician through offline electronic data transfer from the site by the project manager on a monthly basis. The statistician collates the data, maintains the database, and reviews data quality in terms of numbers, consistency and completeness. Measurement of percentage agreement among the data collectors is obtained once a year, to ensure the reliability of the data collected. Several strategies are adopted to achieve adequate participant enrolment including three telephonic follow-up calls to participants not available during in-person visits, two reminders for follow-up visits and rescheduling visits as per participant convenience. Recruitment, follow-up rates, and missing data are discussed at monthly team review meetings between a data manager and data collectors.

### Data storage, security, and confidentiality

Study data is anonymized using unique study identification codes for participants, which is matched to the physical consent form and then entered in the study database. Only the consent form includes personally identifiable details. A code sheet linking the participant’s personal identifiable information is linked to the unique study identification code. Data is stored on a password-protected external hard drive periodically as a back-up. All consent forms and data forms are stored in a locked cabinet at the site office in Mehsana, accessible only to the PI and data manager. After the study is over, the data will be stored in the sealed cabinet as required by Indian regulations.

### Data monitoring

An advisory committee consisting of 4 experts in medical ethics, public health administration and public health and social science research was formed to monitor the implementation and research. The Committee meets every 6 months with the research team and makes periodic site visits to personally interact with a few participants. All adverse events will be reported to the committee.

### Statistical analysis

Baseline characteristics will be summarized using counts (percentages) for categorical variables and means (SD) for continuous variables. The analysis will be based on intention to treat and participants will be analyzed in the group that the cluster was assigned to at each time point.

The analysis plan is based on the Hussey and Hughes model for the analysis of cross-sectional SW-CRCT designs [15]. Generalized Linear Mixed Model (GLMM) will be used to determine the size and direction of the difference between the control and intervention conditions for primary and secondary outcomes. The estimated intervention effect will be reported as the mean outcome difference for continuous variables and Odds Ratio for categorical variables between intervention and control condition assuming a constant treatment effect over time. Estimates of the difference and 95% CIs will be calculated. To take the time effect into account, all analyses will be adjusted for time (periods) of the intervention and clusters. Period (time) and intervention (counseling sessions) will be specified as fixed effects and clusters as a random effect. The analysis will be adjusted for baseline covariates to account for potential imbalance arising due to different recruitment procedures and regional differences across control and intervention conditions. The analysis plan does not include any interim analyses.

Two broad model extensions [41], random cluster by period effect and random cluster by treatment effect will be used for secondary analysis. The secondary analysis will investigate an interaction effect between intervention and time and the interaction effect between cluster and time. Additional analyses of the primary outcome will be conducted controlling for demographic variables if required. Statistical analyses will be carried out using STATA version 14 [38].

### Economic evaluation analysis

All data will be analysed in Microsoft excel. The ratio of costs and benefits will be calculated and will be presented as an ROI. This will inform whether *Atmiyata* intervention is a sound investment. Apart from this, the study will also provide information on the economic burden of CMD in the Mehsana district, which is of value to funders, policymakers and can be used for advocacy purposes. The economic burden of CMD will include direct medical cost (out of pocket expenses on drugs, diagnostics, consultations fees during outpatient visits and hospitalization), direct non-medical cost (out of pocket expenses on transport, food, accommodation while seeking treatment) and indirect cost (time spent by a patient and accompanied persons). As one of the secondary outcomes of the study is to understand the changes in the quality of life of the CMD patient using the EQ-5D, we will conduct a cost-utility analysis as an additional analysis in the economic evaluation. Cost-utility analysis expresses the value for money in terms of a multi-dimensional health outcome. The incremental cost-effectiveness ratio, in this case, is usually expressed as the incremental cost to gain an extra quality-adjusted life-year (QALY) [29]. Consolidated Health Economic Evaluation Reporting Standards (CHEERS) checklist will be followed while reporting the results of ROI and cost-utility analysis [42].

## Discussion

*Atmiyata* is a community-led intervention focused on reducing distress, particularly depression and anxiety symptoms in rural communities in India. The evaluation of the intervention through SW-CRCT, offers several unique opportunities. There is limited literature on SW-CRCTs conducted in low and middle-income countries, particularly involving mental health interventions. This study will contribute to this sparse evidence base and be able to use implementation lessons to inform further scale-up of the intervention to other districts and states, as well as to inform potential intervention scale-up in other settings.

Several challenges exist with using an SW-CRCT design, including a lack of consensus on the model of analysis [41]. Although the *Ottawa Statement* [43] and the Council for International Organizations of Medical Sciences (CIOMS) guidelines [44] provide relevant guidance to the design and conduct of Cluster Randomised Trials, neither document provides guidance specific to SW-CRCT. Other analytical challenges include modelling secular trends, time-varying intervention effect and modelling treatment effect heterogeneity. Different recruitment strategies may confound the statistical analysis. For intervention condition, the sample is drawn from cases identified by Champions. Champions are trained to identify people with CMD, there is a likelihood that they may identify people with higher GHQ scores, and this may confound the analysis and effect. We acknowledge this confounding effect due to different recruitment strategies, and the statistical analysis plan includes adjustment with baseline covariates to account for this confounding. Despite these limitations, we chose this design as being ethical and equitable, as all clusters receive intervention before the end of the trial. In addition, with a control and an intervention period in each cluster, outcomes can be compared within and across clusters which increase statistical power.

Another challenge is the lack of consensus on the cut-off score for depression using GHQ-12. Patel et al. [45] recommended a cutoff score of 7/8 for clinic-based populations. Goldberg et al. [46] suggested the best threshold for GHQ-12 scores varied from 1/2 to 6/7, with the most common cut-off score being 2/3. A recent Indian study confirmed an optimal cut-off score of 2 for community studies based on a receiver operating characteristic curve (ROC) analysis [47]. Taking all the above into account, we conservatively chose 3 as the cutoff score for our community study as it ensures the inclusion of community participants with mild to moderate CMD.

We anticipate challenges during data collection of the trial in a large community setting. Due to stigma and silence around mental ill health, there is a likely reluctance to participate in community mental health studies. Refusal to participate may also be due to the caste, gender, religion, and other social attributes. To address these challenges, data collectors are trained and re-trained on how to build rapport with participants and to maintain privacy and confidentiality. We recruited data collectors with diverse social attributes to represent Mehsana’s heterogeneous population.

Community members often change residence, or the address is incorrectly entered in the electoral register, which increases the time for identification of control condition participants. Data collection timelines also have to accommodate for community events such as farming season, religious festivities. Safety of data collection staff is an equally important concern given the large geographical area being covered. The team will conduct regular meetings to troubleshoot challenges on the field to adhere to the protocol.

Another challenge is to assess the extent to which Champions adhere to the counselling model that they have been trained in. A qualitative method is employed in to assess the fidelity. To do this, we will analyze audio recordings of counselling sessions, assessing a random sample of recordings amounting to 5% of the total counselling sessions delivered by Champions and the results of which will be published separately.

## Trial status

Protocol version: v4, 07 May 2018

Protocol modification in Clinical Trial Registry- 06 April 2018

Date of recruitment- April 2017

Date of recruitment completion- August 2019

We intend to publish trial results in an open-access journal and through meetings with various district and state-level stakeholders.

## Supplementary information

**Additional file 1.** Protocol for Tracking Adverse events.

**Additional file 2.** Gatekeeper consent form.

**Additional file 3.** Consent form for research participant.

## Data Availability

The datasets will be available to appropriate academic parties on request from the principal investigator in accordance with the data sharing policies of the institute within one year of completion of a complete analysis of the data.

## Abbreviations

CF: Community Facilitator
CMD: Common mental disorders
CSQ: Client Satisfaction Questionnaire
DMHP: District Mental Health Programme
EQ-5D: EURO Quality of life- 5D
EUC: Enhanced Usual Care
GAD-7: Generalized Anxiety Disorder 7
GHQ-12: General Health Questionnaire 12
PHC: Primary Health Centre
PHQ-9: Patient Health Questionnaire 9
PI: Principal Investigator
PM: Project Manager
ROI: Return on Investment
SPS: Social Participation Scale
SRQ-20: Self Reporting Questionnaire 20
SW-CRCT: Stepped Wedge Cluster Randomized Controlled Trial
WHO-DAS-12: World Health Organization’s Disability Assessment Scale 12
