# Supplementary material for: Atmiyata, a community-led intervention to address common mental disorders: Study protocol for a stepped wedge cluster randomized controlled trial in rural Gujarat, India
Source: Trials. 2020 Feb 21;21:212. doi: 10.1186/s13063-020-4133-6 (PMC7035701; doi:10.1186/s13063-020-4133-6)
Supplement: Supplementary file 1 — Additional file 1. Protocol for Tracking Adverse events. [file 13063_2020_4133_MOESM1_ESM.pdf]

Protocol for Tracking Adverse events

Even though the possibility of adverse events which directly related to the intervention are low, the project team will maintain an incident register to record AE.

What is an adverse event?

An AE is an unintended occurrence experienced by a study participant which: results in death; or is life-threatening;

Attempted suicide / Self harm and Suicide are considered as AE. Suicide intent, self-harm and overdose are highly unlikely, but are associated with common mental disorders and therefore would be an expected AE. We do not expect treatment to cause the above events. Therefore, if any of the following AEs are reported or observed then the AE procedure should be implemented as soon as participant safety has been assured.

1. Self-harm

a) If a person exhibits any signs of self-harm they will be encouraged to contact health practitioner at Government facility on same day. In the event, that they refuse to seek help and their safety is a concern, then champion should notify this to community facilitator and community facilitator will try to talk to the person and the family and encourage them to visit the nearest health facility.

2. Overdose

a) If the person reports or exhibits signs of excessive drug consumption/ intention to overdose they will be encouraged to contact health practitioner that day.

b) If they refuse to seek help and their safety is a concern, then champion should inform community facilitator within 48 hours who in turn try to talk to the person and the family and encourage them to visit the nearest health facility

Reporting Adverse Events (AEs)

For reporting, we will record the village and vaas (subsection of village) name, identification number, date of reporting, date of event, description of the event, relationship to the intervention. The relatedness of the event to the intervention will be assessed by an independent psychiatrist.

The community Facilitator should inform Project manager(PM) about the event within 24 hours. The PM in consultation with project lead, will make a report and submit it to the Ethics Committee within 15 days.

Life threatening or hospitalising medical diagnoses/assessments related to physical co-morbidities are considered expected and unrelated AEs and do not need reporting. These will be recorded in supervision and monitoring form.

Recognition of AEs will be included in the training of all research team members in Gujarat. This training will include-: definition of adverse events, explanation of the adverse events in the local context, how to detect AEs, and a series of steps to follow once an AE occurs.

Any participant who have encountered an adverse event will be followed for six-month period and encouraged to seek help from public health facility if necessary.

<u>Mitigation strategies-</u>

- The research team will be trained in recognizing and addressing any unintended adverse emotional effects during outcome assessments or during delivery of the intervention.
- All interviews will be conducted, to the extent possible, in a private setting.
- Any participant in an acute crisis (e.g. those who have made a suicide attempt) will be identified as a high-risk case and referred to specialized care, as per the referral pathways mentioned earlier.

<u>Protocol for reporting a serious adverse event:</u>

The following steps must be taken:

- The champion must inform Community Facilitator (CF) within 48 hours.
- The CF must inform project manager within 24 hours.
- Encourage the person to seek medical help.
- If the person refuses to seek help, the family members need to be spoken to and encouraged to seek help for the person.
- Project Manager (PM) needs to record details the details of the event.
- PM will take consultation from independent psychiatrist.
- PM/ PI will submit this report to ethics committee within 15 days.
