# Supplementary material for: Atmiyata, a community-led intervention to address common mental disorders: Study protocol for a stepped wedge cluster randomized controlled trial in rural Gujarat, India
Source: Trials. 2020 Feb 21;21:212. doi: 10.1186/s13063-020-4133-6 (PMC7035701; doi:10.1186/s13063-020-4133-6)
Supplement: Supplementary file 2 — Additional file 2. Gatekeeper consent form. [file 13063_2020_4133_MOESM2_ESM.pdf]

Gatekeeper (village head, community leaders) consent form-

About the Project-

Atmiyata project is a community-based mental health intervention focused on promoting wellness and reducing distress through community volunteers in a rural India. The project has two components- Research and Implementation. I will be focusing on Research.

The project is being implemented by Indian Law Society (ILS), Pune in collaboration with the Department of Health and Family Welfare, Govt. of Gujarat Gujarat and Altruist NGO, Ahmedabad.

Research description

Atmiyata study will be implemented across 500 villages in Mehsana district of Gujarat from April 2017 to August 2019. The data collection will be done through random sampling from these villages in Mehsana District.

Procedures and Protocol

For the purpose of the study, we will be interviewing a few members of your village on issues related to stress and distress.

The participant will be asked questions about their background, life experiences, mental health, the mental health care available and its cost. If some of the topics we discuss appear too personal or difficult to discuss they have the right to stop the interview at any time, or to skip any questions that you don't want to answer. The data is collected in electronic format using tablets, it will be kept secure and anonymous.

FAQs-

1. Why is the study being conducted in our village?

The Atmiyata study in being conducted across all the villages in the District of Mehsana, your village was chosen based on random sampling method. We did not choose your village intentionally.

2. What are the benefits and risks involved?

Participation in the study is completely voluntary. Their participation will help health policymakers learn how to provide mental health services that can reach many more people. Also, it will provide information on cost required for existing mental health care.

3. What happens with data collected?

● The information gathered from the interviews will be kept strictly confidential.
names of the participants will be not be recorded on the electronic form. The consent form will be kept in a locked cabinet, a copy of the consent form will remain with the respondent.

4. **Whom to call in case of an emergency**? **Who do you call if you have questions or problems?**

● You can contact Dr Kaustubh Joag, (Principal Investigator) on this mobile number 9881769500 if you have questions about your participation in this study or for any other questions about the study.

5. Where can I get more information regarding the training of volunteers?

An Atmiyata Community Facilitator will visit your village in the course of two years and provide more information regarding selection and training of community volunteers.

Do you grant permission for the research study in your village? ______ Yes ________ No

Declaration of the Researcher
I have explained the above considerations in a language that the person understands, regarding their community's participation in the study. There was space given to ask any questions and have been answered to the best of my abilities.

Signature of the researcher _________________

Date ______________
