# Supplementary material for: Atmiyata, a community-led intervention to address common mental disorders: Study protocol for a stepped wedge cluster randomized controlled trial in rural Gujarat, India
Source: Trials. 2020 Feb 21;21:212. doi: 10.1186/s13063-020-4133-6 (PMC7035701; doi:10.1186/s13063-020-4133-6)
Supplement: Supplementary file 3 — Additional file 3. Consent form for research participant. [file 13063_2020_4133_MOESM3_ESM.pdf]

Consent Form for research participants

ATMIYATA Study: Promoting Well-being, reducing Distress and Improving access to mental health care through self-help groups and farmers clubs in rural Gujarat

ID Number: ______________________

Principal Investigator: Kaustubh Joag

Purpose of the Study

Hello. My name is _________. I work as a Research Assistant with Indian Law Society (ILS), Pune. ILS is implementing a project called Atmiyata. The project focuses on improving access to mental health care and improving wellbeing of community. The project will be implemented across all villages in the district of Mehesana, Gujarat and the data collection will be done in random sample from these villages in Mehesana District

Procedures and Protocol

We would like to interview you which will take 45 mins to one hour.

If you agree to participate in this study, we would like to meet you to interview you in a private setting of your choice, where you will feel comfortable to talk with us. During the interview, you will be asked questions about your background, your life experiences, your mental health, the mental health care available and its cost. If some of the topics we discuss appear too personal or difficult to discuss you have the right to stop the interview at any time, or to skip any questions you don't want to answer.

Benefits and Risks

Your participation is completely voluntary. If you participate, your participation will help health policy makers learn how to provide mental health services that can reach many more people. Also your participation will provide information about cost needed for existing mental health care.

Confidentiality

Whatever information you will provide will be kept totally confidential.

Your name will be not be recorded on the interview form or on any other data forms. The interview schedules will be kept in a locked cabinet. We will keep a record of who participates in this study, but we will keep that information separate from the data forms, so nobody would be able to link your names to the information you provide.

Whom to call in case of an emergency? Who do you call if you have questions or problems?

- You can contact Dr Kaustubh Joag, (Principal Investigator):0-9881769500 if you have questions about your rights as a study participant or if you have any questions or objections about the study procedure and content.

Do you agree to be interviewed? ______ Yes ________ No

Subject's Statement

I have read or have been read the above considerations regarding participation in the study. I have been given a chance to ask any questions I may have and any questions have been answered to my satisfaction.

I understand that my records will be kept private and that I can stop the interview at any time. I also understand that my decision to stop the interview will not affect the health services I receive.

NOTE WHETHER RESPONDENT AGREES TO BE INTERVIEWED

[ ] DOES NOT AGREE TO BE INTERVIEWED -->THANK PARTICIPANT FOR HER TIME

[ ] AGREES TO BE INTERVIEWED

[ ] AGREES TO BE INTERVIEWED. RESPONDENT IS NON-LITERATE & HAS GIVEN VERBAL CONSENT

Signature _____________________ Date_________________________

Place _______________________

Name of the Participant: ____ ___________________

Son / Daughter / Spouse of: _____________________________________ ____________________

Complete postal address: _____________________________________ ____________________

____

Contact Number(s):

Thumb impression (for illiterate individual) – _________________

Date ________________ Place___________________

Witness present at the time of consent – (For Illiterate individuals) – Yes / No

Witness signature – Date:

Place:

Please tell me if this time and place are good to talk?

If there are any problems we can agree on a place and time of your choice.

Investigator's statement

I, the undersigned, have explained to the volunteer in a language he understands the procedures to be followed in the study and the risks and benefits involved.

_____________________________ ___________________

Interviewer's signature Date

Interviewers name:

Phone Number:
